# CROSS-CULTURAL VALIDATION OF THE OARS INSTRUMENT FOR THE ASSESSMENT OF ELDERLY ANGOLANS

**DOI:** 10.1093/geroni/igad104.3255

**Published:** 2023-12-21

**Authors:** Rita Barbara

**Affiliations:** Universidade de Aveiro, Sao Paulo, Sao Paulo, Brazil

## Abstract

**Background:**

The Older Americans Resources and Services (OARS) instrument is widely used internationally, but requires validation in specific contexts, such as Angola. This study aims to cross-culturally validate the OARS for assessing elderly Angolans.

**Methods:**

The cognitive and functional domains of the OARS were cross-culturally adapted, followed by psychometric analysis on a representative sample. Domains of activities of daily living and mental health were evaluated. Internal reliability was assessed using Cronbach’s alpha coefficient, resulting in 0.80 for activities of daily living and 0.80 for mental health, with a 95% confidence interval.

**Results:**

The psychometric analysis demonstrated consistent reliability in the domains of activities of daily living and mental health, with Cronbach’s alpha coefficients of 0.80 for both, supported by 95% confidence intervals. These results indicate substantial reliability.

**Conclusion:**

The cross-cultural validation of the OARS is ongoing, and preliminary results from the psychometric analysis reinforce the reliability of the domains of activities of daily living and mental health. Adapting the OARS could be a valuable tool for assessing elderly Angolans, providing reliable and internationally comparable data. Keywords: OARS, elderly, cross-cultural validation, psychometric analysis.

